# The tangential view described by Moneim to demonstrate scapholunate dissociation: an update

**DOI:** 10.1007/s00590-022-03391-z

**Published:** 2022-09-16

**Authors:** Allicia O. Imada, Kathryn Welch, Gary Mlady, Moheb S. A. Moneim

**Affiliations:** 1 Department of Orthopaedics & Rehabilitation, The University of New Mexico, Albuquerque, NM 87121 USA; 2 Department of Radiology, The University of New Mexico, Albuquerque, NM USA

**Keywords:** Tangential, Wrist, Scapholunate, Radiograph

## Abstract

**Purpose:**

Scapholunate dissociation is a common and significant injury to the wrist. Radiographs are important in the diagnosis of this injury and in the planning of treatment. The tangential radiograph view was described almost 40 years ago as a method for accurately measuring scapholunate gaps. The hand is positioned on a 20° foam rubber block and the thumb on the cassette, which positions the scaphoid and lunate articular surfaces parallel, without patient discomfort or effort. The goal of this study was to review this method with more recent data and in a larger group of patients.

**Methods:**

Radiographs of 31 patients who had scapholunate interosseous ligament tears and surgical repair over a 9 year period were retrospectively evaluated. Each of the four authors independently measured scapholunate gaps for posteroanterior and tangential views.

**Results:**

The tangential view gaps were significantly greater than the posteroanterior gaps overall. Similar results were found for borderline cases where the posteroanterior gap was less than 3 mm. Every tangential view gap measurement was greater than its respective posteroanterior gap with good inter-rater reliability.

**Conclusion:**

The tangential view is a reliable radiographic method to identify scapholunate gaps. It should be obtained when there is clinical concern for scapholunate dissociation, especially in patients with borderline posteroanterior gaps.

## Background

Scapholunate (SL) injuries are the most common cause of intercalary carpal instability and are often encountered by hand surgeons and radiologists [1, 2]. The scapholunate interosseous ligament (SLIL) primarily stabilizes the SL joint [3, 4]. Complete tearing and attenuation of secondary stabilizers lead to gapping of the SL interval on radiographs, eventually leading to scapholunate advanced collapse [5].

Plain radiographs are accessible, relatively inexpensive, and noninvasive methods of evaluating wrists for SL injuries [6]. Neutral posteroanterior (PA) and lateral radiographs are often taken to evaluate widened SL gaps (greater than 2 mm) [5–8]. The scaphoid and lunate articular surfaces overlap on routine PA views of the wrist, which can make it difficult to accurately measure SL gaps. Various stress views have also been described, but these can be painful and rely heavily on patient effort [6, 7, 9].

In 1981, Moneim described the tangential view in the Journal of Bone and Joint Surgery [10]. For this view, the patient positions their hand with the index finger resting on a 20° foam rubber block and the thumb on the cassette. By doing this, the scaphoid and lunate articular surfaces are parallel to the beam. The normal gap of the tangential view ranged from 0 to 1.6 mm, and a 2 mm gap on the tangential view was considered the upper limit of normal.

We sought to follow up on the Moneim study published almost 40 years ago by retrospectively evaluating radiographs of patients who underwent SLIL open repair procedures to validate this method. We hypothesized that the tangential view reliably identified SL gaps more clearly than the PA view.

## Methods

**Fig. 1:**
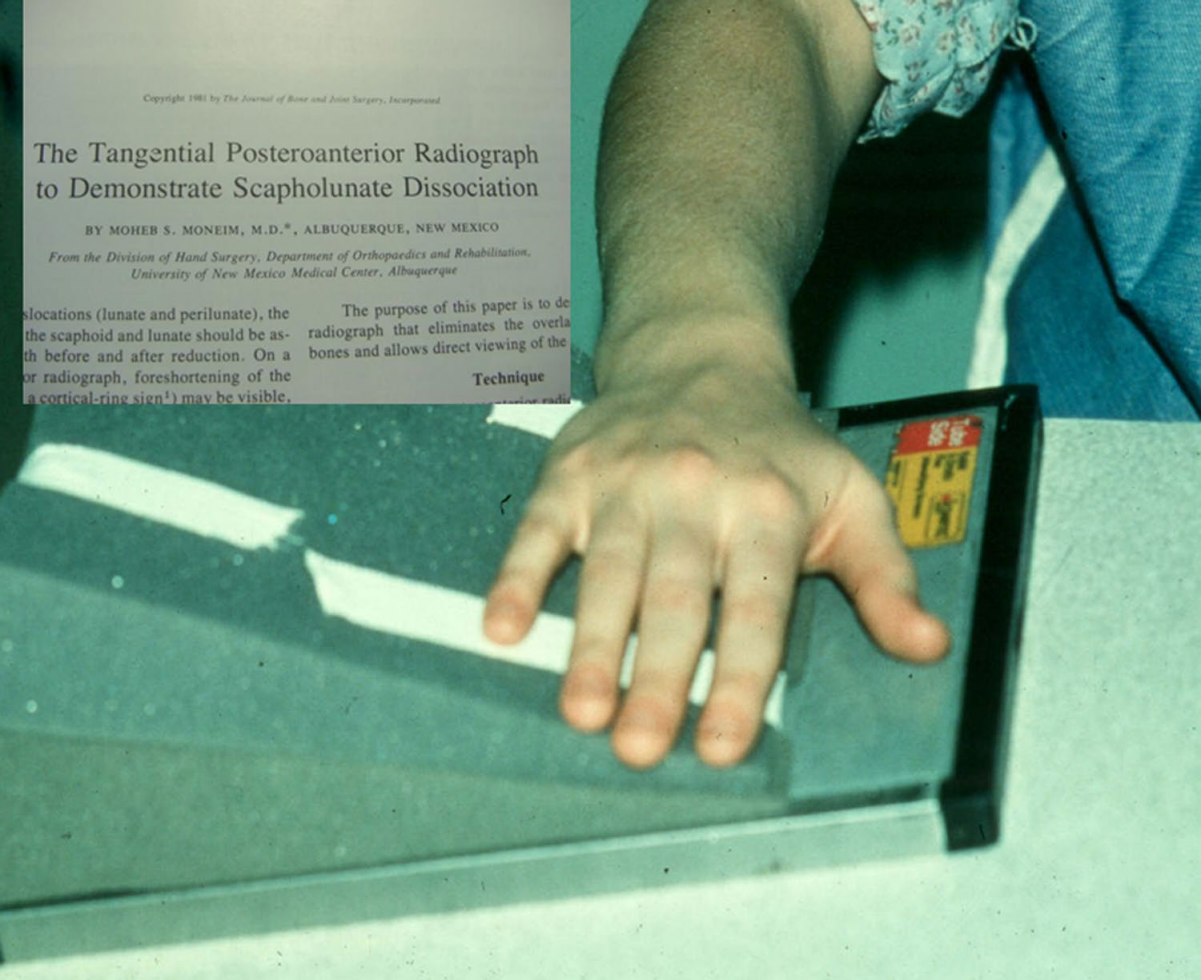
Patient positioning as described by Moneim in 1981. The patient will rest their hand on a 20° foam rubber block. The index finger rests on the block while the thumb rests on the radiograph cassette. Reprinted with permission from Moheb Moneim 1981

This study received approval from the Institutional Review Board. A retrospective radiographic review was completed for all patients who underwent SLIL repair procedures for static SLD between March 1, 2006, and March 31, 2015, at a single level-one trauma center. All patients had intraoperatively confirmed SL dissociation (SLD) with SLIL complete tears or laxity with scarring. Preoperative radiographs, including PA and tangential views, were done. If contralateral radiographs were available, they were also included for comparison. Patients were positioned as shown in Fig. 1 as previously described by Moneim. Preoperative non-contrasted magnetic resonance imaging (MRI) was examined if available. No preoperative wrist arthroscopy was done. Exclusion criteria included previous wrist procedures or participation in Moneim original study. Patient age, time from injury to surgery, and final follow-up were recorded.

**Fig. 2:**
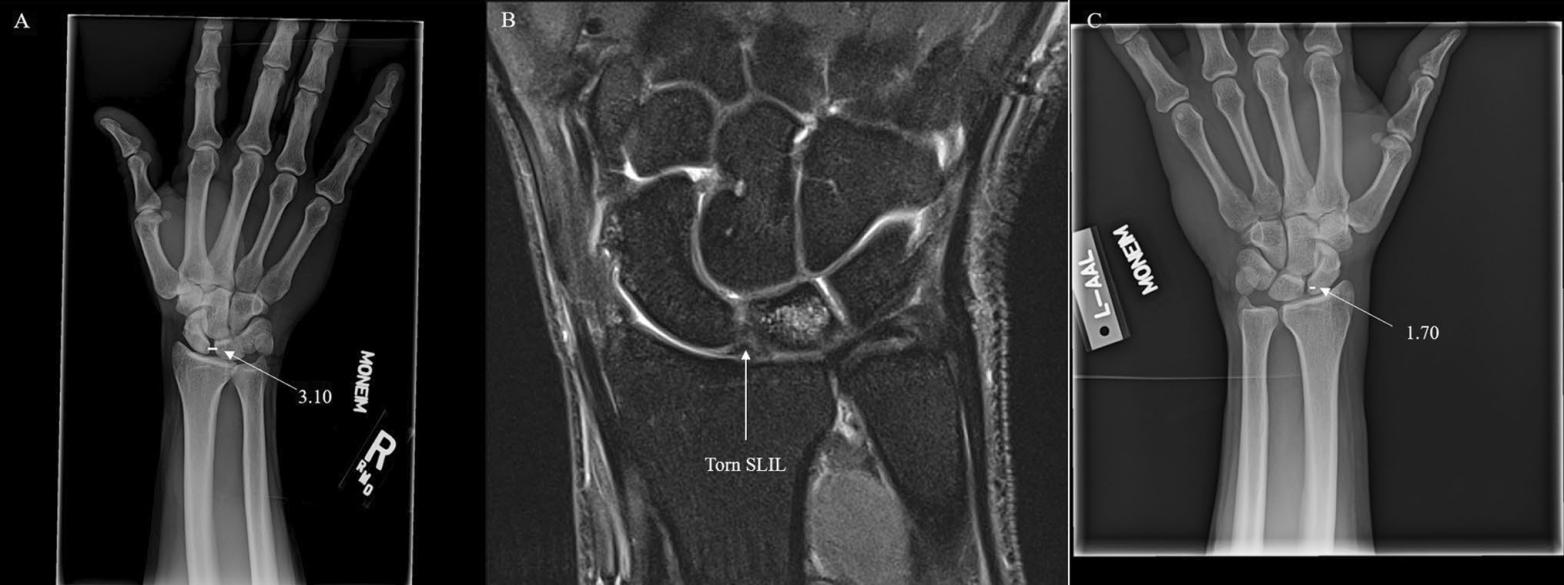
**A**-Radiograph of tangential Moneim view of injured wrist with average SL gap of 3.10 mm. **B**: Coronal Proton Density Turbo Spin Echo (PD TSE) MRI showing torn SLIL. **C**-Radiograph of tangential Moneim view of normal wrist with average SL gap of 1.70 mm

**Fig. 3:**
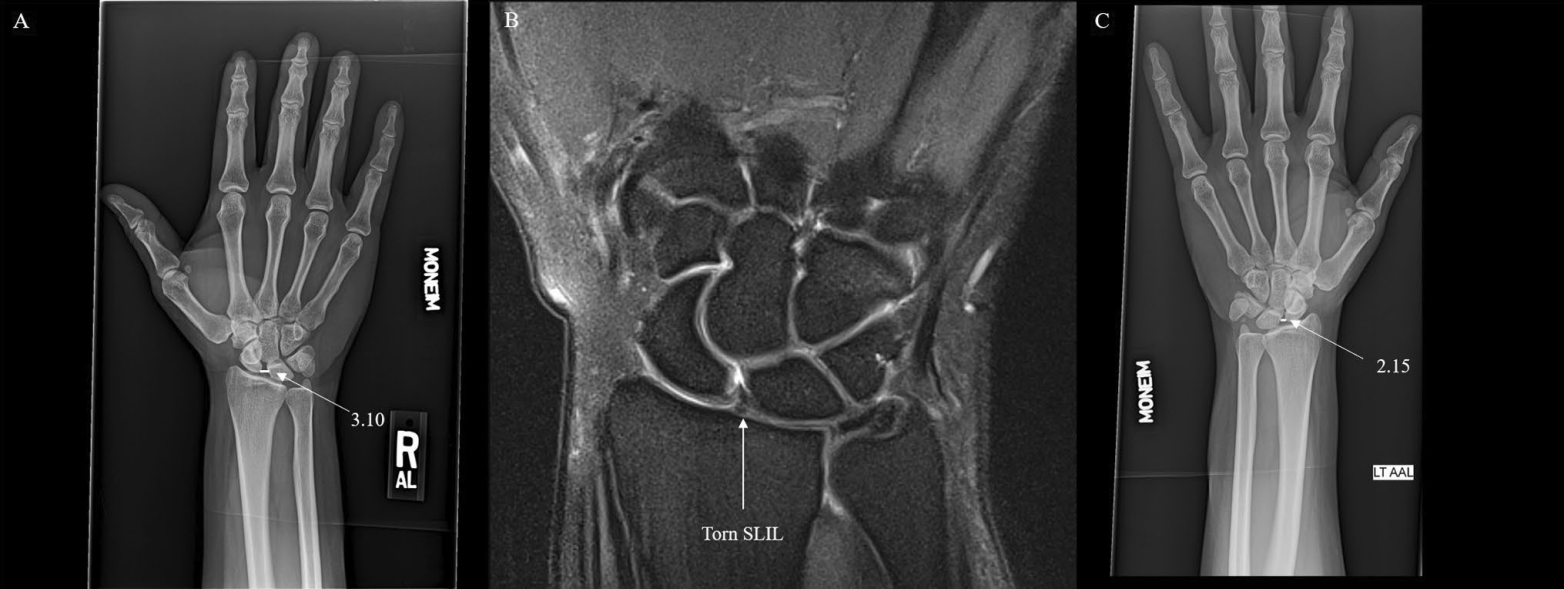
**A**-Radiograph of tangential Moneim view of injured wrist with average SL gap of 3.10 mm. **B**: Coronal Proton Density Turbo Spin Echo (PD TSE) MRI showing torn SLIL. **C**-Radiograph of tangential Moneim view of normal wrist with average SL gap of 2.15 mm

**Fig. 4:**
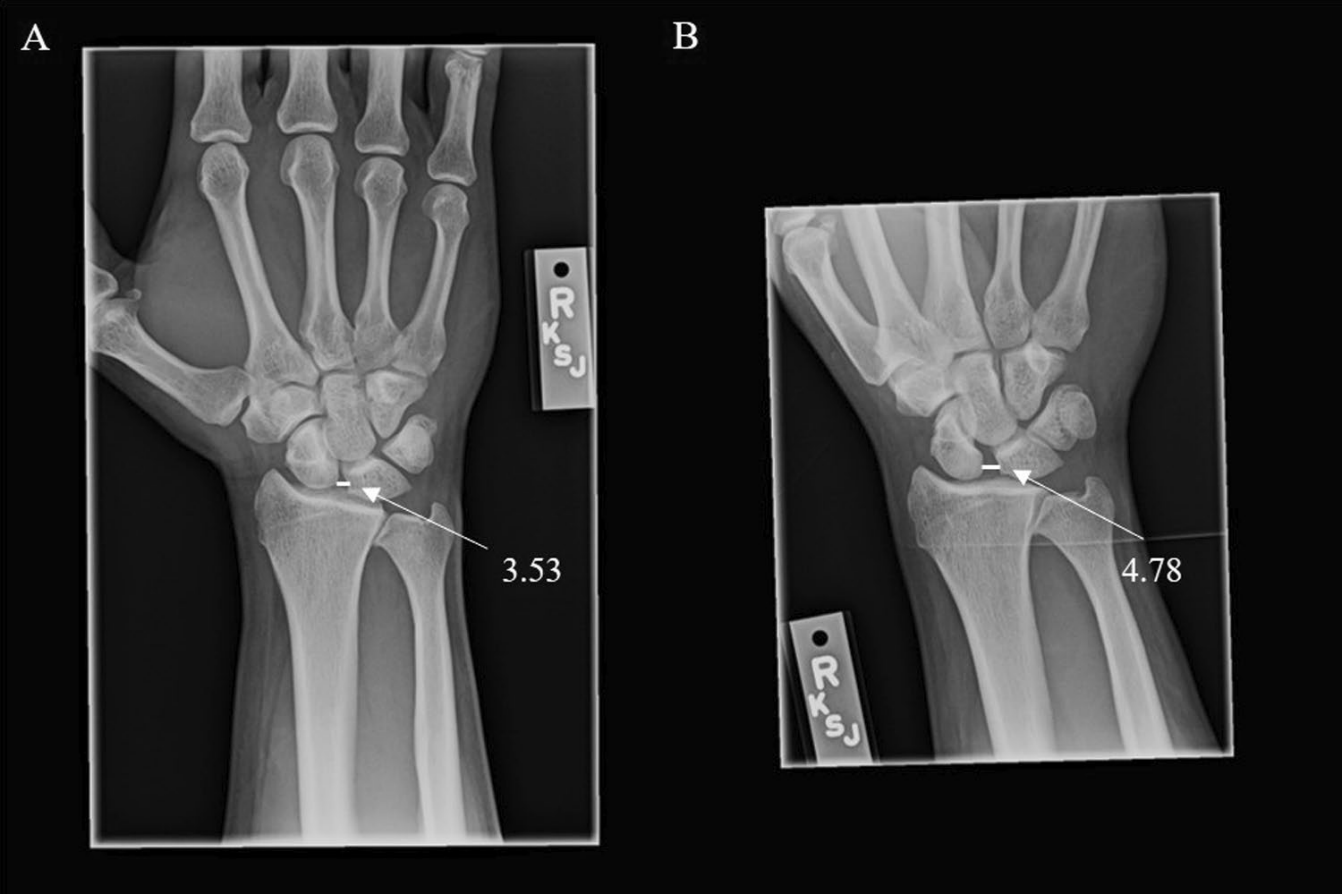
Radiograph of PA (**A**) and tangential Moneim view (**B**) with average SL gaps of 3.53 mm and 4.78 mm at 12 years from injury. See radiocarpal degenerative changes, confirmed at surgery

Preoperative SL gap was measured independently by each of the four authors (two orthopaedic surgeons and two radiologists) on PA and tangential views using the Philips IntelliSpace PACS 4.4 measuring tool (Figs. 2, 3, 4). The tangential view in cases with multiple PA views or unlabeled radiographs was the radiograph which showed a clear space between the capitate and hamate with no overlap.

RStudio statistical software was used for analysis. Scatter plots, paired *t*-tests, and linear regression models were used to compare the tangential view and PA gaps. Inter-rater reliability was measured using intra-class correlation (ICC) estimates, and 95% confidence intervals were calculated with the *icc* function of the *irr* package in R. The model was two-way, absolute-agreement, and single-measurement per rater. Radiographs with mean PA gaps of less than 3 mm were also analyzed as a separate group using the same tests to examine this subset with borderline gaps. A similar statistical analysis was used for the normal tangential radiographs.

## Results

Thirty-one patients were identified, 21 men and 10 women. Ages ranged from 18 to 70 years old. Time from injury to surgery ranged from 1 week to 14 years (Tables 1 and 2). Seven patients had acute injuries, at or less than five weeks, while the majority had injuries between 4 and 8 months. Four patients had chronic injuries over 18 months. Follow-up ranged from 1 to 14 months postoperatively. Nineteen patients had preoperative MRIs confirming SLIL tears.

**Table 1:** Mean posteroanterior and tangential gap measurements

Patient	History of injury	Mean PA ± SD	Mean tangential ± SD
1	8 M	4.65 ± 1.46	9.60 ± 1.16
^+^2	5 W	4.35 ± 0.66	5.05 ± 0.80
3	3 W	3.83 + 0.55	4.50 ± 0.33
4	5 W	3.93 ± 0.65	7.35 ± 1.03
5	6 M	4.73 ± 0.41	6.00 ± 0.46
6	1 W	4.15 ± 0.92	4.53 ± 0.49
7	3 M	5.10 ± 0.27	7.55 ± 0.38
8	4 M	3.03 ± 0.29	4.20 ± 0.56
9	2 M	4.93 ± 0.55	5.55 ± 0.91
10	7 M	3.63 ± 0.48	4.70 ± 0.31
11	7 M	3.95 ± 0.56	5.00 ± 0.43
^+^12	4 W	3.20 ± 0.21	4.85 ± 0.36
13	14 Y	3.15 ± 0.15	4.20 ± 0.70
14	1 W	4.93 ± 0.65	6.23 + 0.54
15	5 M	3.25 ± 0.45	4.15 + 0.30
16	19 M	4.73 ± 0.42	6.78 + 0.48
17	7 M	5.20 ± 0.36	6.05 ± 0.21
18	2 M	5.73 ± 0.24	7.78 ± 0.33
19	12 Y	3.53 ± 0.35	4.78 ± 0.25
^+^20	5 M	3.08 ± 0.48	3.48 ± 0.38
21	5 M	3.83 ± 0.35	5.43 ± 0.18
22	4 M	4.38 ± 1.21	6.03 ± 1.60

Measured in millimeters (mm) averaged over the four raters in patients with PA gaps greater than 3 mm. History of injury is the time from injury to surgery

*SD* standard deviation, *M* months, *W* weeks, *Y* years, *PA* posteroanterior

^+^Available contralateral preoperative tangential radiograph

**Table 2:** Mean posteroanterior and tangential view gap measurements for borderline cases, with PA view gaps less than 3 mm and larger tangential view gaps

Patient	History of injury	Mean PA ± SD	Mean tangential ± SD
23	3 M	1.85 ± 0.35	5.53 ± 0.52
^+^24	5 M	2.50 ± 0.23	6.05 ± 0.71
^+^25	7 M	1.48 ± 0.66	2.90 ± 0.30
^+^26	3 M	1.60 ± 0.34	3.35 ± 0.29
27	4 W	2.73 ± 0.49	7.05 ± 0.62
^+^28	5 M	1.80 ± 0.49	3.10 ± 0.19
^+^29	2 Y	2.95 ± 0.59	3.03 ± 0.43
^+^30	3 M	1.65 ± 0.45	3.10 + 0.70
31	5 M	2.53 ± 0.36	4.55 ± 0.69

*SD* standard deviation, *M* months, *W* weeks, *Y* years, *PA* posteroanterior

^+^Available contralateral preoperative tangential radiograph

**Fig. 5:**
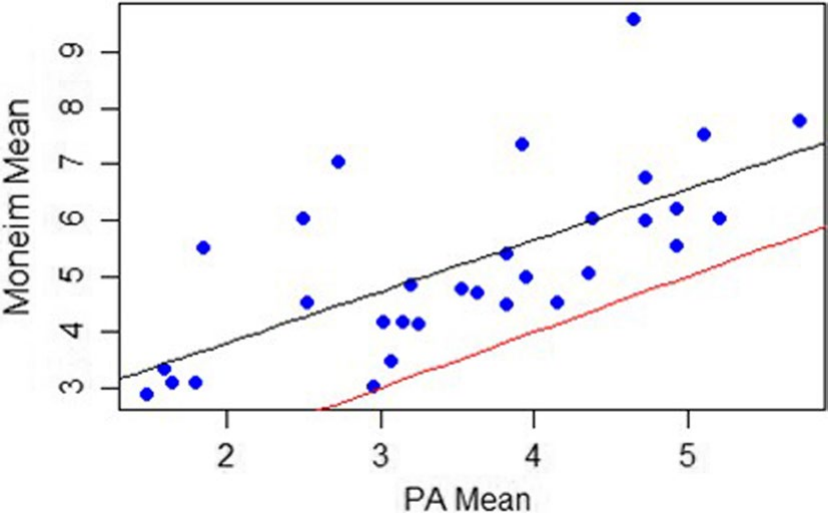
Scatterplot and linear regression model of tangential view and posteroanterior mean gaps in millimeters. Each patient’s mean gap measurement over the four raters represents one of the dots. The black line is the best fit using a linear model. The red line represents *y* = x and shows that every tangential measure is larger than the posteroanterior measure

When averaged over the four raters, the mean gap measurement for the tangential view was significantly greater than its respective PA measurement. The means were 3.56 mm and 5.24 mm for the PA group and tangential group, respectively, which were significantly different (Tables 1 and 2) (*P* = 7.34e-09, mean of differences − 1.68). The 95% confidence interval for the mean of the differences was − 2.11 to − 1.25. A scatterplot with a linear regression model is shown in Fig. 5. Both the coefficients of the model and overall model are significant (*P* = 2.9e-05).

When the subset group of nine borderline radiographs with a mean PA gap of less than three mm was examined, the tangential view gaps were significantly greater (*P* = 0.001, mean of the differences − 2.175) (Table 2). The 95% confidence interval for the mean of the differences is − 3.24 to − 1.11.

Inter-rater reliability was tested and showed good reliability between raters with ICCs of 0.75 for the PA view and 0.83 for the tangential view gaps (*P* < 0.001 for both groups) [11].

Preoperative tangential radiographs of the contralateral wrists were available for nine patients with a mean gap of 2.3 mm (Table 3). Inter-rater reliability was poor with an ICC of 0.39, with *P* = 0.002 showing that measurements were close enough that they are unlikely to be random.

**Table 3:** Mean tangential view gap measurements of normal contralateral wrist and injured wrists

Patient	SLD mean tangential ± SD	Normal mean tangential ± SD
2	5.05 ± 0.80	2.98 ± 0.73
24	6.05 ± 0.71	2.33 ± 0.41
25	2.90 ± 0.30	2.23 + 0.25
26	3.35 ± 0.29	2.30 + 0.37
12	4.85 ± 0.36	2.65 + 0.35
28	3.10 ± 0.19	2.15 ± 0.36
29	3.03 ± 0.43	1.93 + 0.26
30	3.10 + 0.70	1.70 ± 0.14
20	3.48 ± 0.38	1.85 ± 0.29

*SD* standard deviation, *SLD* scapholunate dissociation

## Discussion

SL dissociation is the most common form of carpal instability and can be treated with various repair or stabilization procedures [12–14]. Radiographs to identify the SL gap are important in the diagnosis and treatment of SL dissociation and standard PA radiographs may not accurately show this SL gap due to overlap between the scaphoid and lunate articular surfaces. Our study showed that the tangential view showed significantly increased SL gap measurements, even in the borderline patients with PA gaps of less than 3 mm, with good inter-rate reliability when compared to standard PA gap measurements. Contralateral wrist tangential radiographs showed a gap of 2.3 mm, which may be considered the upper limit of normal.

Limitations of this study include those inherent to retrospective studies. There was also the possibility of inherent bias in rater measurements, but we tried to mitigate these with four separate rater-independent measurements. Inter-rater reliability was good, so we felt that measurements were accurate. Inter-rater reliability was poor for normal wrists, but the sample size was small and the *P*-value was significant (*P* = 0.002) showing that these measurements were not likely to be random.

Our results confirm Moneim’s findings and may be especially beneficial to identify static SLD since the method involves no patient active effort [10]. Dynamic radiographs such as the clenched pencil view have been shown to reliably identify SLIL injuries, but can be painful for patients [7]. A study by Patel et al. [15] showed that the clenched fist and PA ulnar deviation views equally diagnosed dynamic instability most effectively, while none of the stress views were significantly better at showing static SL dissociation.

We recommend using 2.3 mm as the upper limit of normal for identifying static SLD on tangential view radiographs based on our mean contralateral wrist measurements. This is consistent with the original Moneim study which noted a cut-off of 2 mm [10]. Two or sometimes three mm gaps have been described in the literature as the upper limit of normal PA views [6, 9, 15]. If there is still concern with normal tangential gap measurements contralateral wrist radiographs, MRI with or without arthrogram, or arthroscopy can be considered [4, 16, 17].

For almost 40 years, the tangential view has been used to illustrate the SL gap in cases of SLD to indicate a tear of the SLIL at surgical exploration. The tangential view requires minimal effort by the patient, whereas other replicated views may lead to discomfort. At our institution, we use the tangential view without modification, as described in the original Moneim publication.

## Conclusions

We have shown with updated data that the tangential view significantly identified larger SL gaps than the standard PA view even in chronic injuries when wrist stiffness or pain may prevent dynamic views. Surgeons should consider obtaining this radiograph when there is suspicion of; lop; op SL dissociation, especially in patients with borderline SL gaps on PA view around 2 to 3 mm.
